# Does Tap Water Quality Compromise the Production of *Aedes* Mosquitoes in Genetic Control Projects?

**DOI:** 10.3390/insects12010057

**Published:** 2021-01-12

**Authors:** Wadaka Mamai, Hamidou Maiga, Nanwintoum Sévérin Bimbilé Somda, Thomas Wallner, Odet Bueno Masso, Christian Resch, Hanano Yamada, Jérémy Bouyer

**Affiliations:** 1 Insect Pest Control Laboratory, Joint FAO/IAEA Division of Nuclear Techniques in Food and Agriculture, Vienna, Austria; h.maiga@iaea.org (H.M.); N.S.Bimbile-Somda@iaea.org (N.S.B.S.); T.Wallner@iaea.org (T.W.); odetbueno123@hotmail.com (O.B.M.); H.Yamada@iaea.org (H.Y.); J.Bouyer@iaea.org (J.B.); 2 Institut de Recherche Agricole pour le Développement (IRAD), PO. Box 2123 Yaoundé, Cameroon; 3 Institut de Recherche en Sciences de la Santé/Direction Régionale de l’Ouest (IRSS/DRO), 01 PO. Box 545 Bobo-Dioulasso, Burkina Faso; 4 Laboratoire d’Entomologie Fondamentale et Appliquée (LEFA), Université Joseph Ki-Zerbo, 03 PO. Box 7021 Ouagadougou, Burkina Faso; 5 Soil and Water Management and Crop Nutrition Laboratory, Joint FAO/IAEA Division of Nuclear Techniques in Food and Agriculture, Vienna, Austria; CH.Resch@iaea.org

**Keywords:** water quality, *Aedes* mosquitoes, vectors, water hardness, electrical conductivity, ions

## Abstract

**Simple Summary:**

Scientists all over the world are continually rearing and producing insects in laboratories for many purposes including pest control programmes. *Aedes aegypti* and *Ae. albopictus* are mosquitoes of public health importance due to their ability to vector human and animal pathogens and thus vector control represents an important component of many disease control programmes. Water is a factor of great importance in the larval environment of mosquito species. However, obtaining sufficient water of reliable quality for mosquito rearing is still challenging, especially in developing and least developed countries, where access even to clean drinking water is limited. In prospect of cost-effective methods for improved mass-rearing toward SIT application, we assessed the impact of using tap water on the development and quality of *Aedes* mosquitoes. Results showed that, tap water with hardness/electrical conductivity beyond certain levels (140 mg/l CaCO_3_ or 368 µS/cm) was shown to have a negative impact on the production of *Ae. albopictus* and *Ae. aegypti* mosquitoes. These results suggest that the quality of water should be checked when using for rearing mosquitoes for release purposes in order to optimize the production performance of mass-rearing facilities. This may have important implications for the implementation of the sterile insect technique in areas where reverse osmosis water is a scarce or costly resource.

**Abstract:**

A mosquito’s life cycle includes an aquatic phase. Water quality is therefore an important determinant of whether or not the female mosquitoes will lay their eggs and the resulting immature stages will survive and successfully complete their development to the adult stage. In response to variations in laboratory rearing outputs, there is a need to investigate the effect of tap water (TW) (in relation to water hardness and electrical conductivity) on mosquito development, productivity and resulting adult quality. In this study, we compared the respective responses of *Aedes aegypti* and *Ae. albopictus* to different water hardness/electrical conductivity. First-instar larvae were reared in either 100% water purified through reverse osmosis (ROW) (low water hardness/electrical conductivity), 100% TW (high water hardness/electrical conductivity) or a 80:20, 50:50, 20:80 mix of ROW and TW. The immature development time, pupation rate, adult emergence, body size, and longevity were determined. Overall, TW (with higher hardness and electrical conductivity) was associated with increased time to pupation, decreased pupal production, female body size in both species and longevity in *Ae. albopictus* only. However, *Ae. albopictus* was more sensitive to high water hardness/EC than *Ae. aegypti*. Moreover, in all water hardness/electrical conductivity levels tested, *Ae. aegypti* developed faster than *Ae. albopictus*. Conversely, *Ae. albopictus* adults survived longer than *Ae. aegypti*. These results imply that water with hardness of more than 140 mg/l CaCO_3_ or electrical conductivity more than 368 µS/cm cannot be recommended for the optimal rearing of *Aedes* mosquitoes and highlight the need to consider the level of water hardness/electrical conductivity when rearing *Aedes* mosquitoes for release purposes.

## 1. Introduction

All organisms are directly or indirectly affected by the physico-chemical attributes of the environment in which they develop [1]. A mosquito’s life cycle includes an aquatic phase. They require water bodies for oviposition and completing the larval and pupal stages. Therefore, water is an important determinant for oviposition, developmental success of immature stages [2] and adult life traits [3]. Extensive literature exists across diverse insect taxa describing the influence of physico-chemical parameters of breeding sites in relation to their abundance, and each species has its preferred water bodies. *Aedes* *aegypti* lay eggs in rainwater generally in artificial containers which has a very low hardness and is similar to reverse osmosis water. Several studies have found that dissolved oxygen, pH, temperature, conductivity and vegetation seem to be driving variables for larval abundance of several mosquito species [4,5,6,7]. In various laboratories all over the world, scientists are continually rearing and producing insects for many scientific purposes and for pest control programmes [8,9,10]. However, the rearing of mosquitoes is complex and demands careful assessment of water quality, larval density, nutrition and environmental conditions. Although most insectaries use deionized or ROW for rearing mosquitoes, many countries located in arid zones often use other water sources including TW [11], surface water, groundwater, and desalinated water for rearing mosquitoes. Various parameters such as water hardness, electrical conductivity, salinity and total dissolved solids are commonly used as indicators of water quality [12,13,14]. The total hardness is the measured content of all divalent cations in the water. Traditionally, it is a measurement of the capacity of water to react with soap and describes the ability of water to bind soap to form lather, which affects the washing process. Calcium (Ca^2+^) and magnesium (Mg^2+^) are the main contributors to the total hardness in most freshwater systems [15,16]. Salts that dissolve in water break into positively and negatively charged ions. In this regard, salinity is a measure of the amount of salts in the water, while the electrical conductivity a parameter used to estimate the level of dissolved salts in water refers to the ability of the material to allow the flow of an electric current, which is carried by ions in the solution [17]. Therefore, high conductivity indicates high water mineralization [18]. Because dissolved ions increase salinity as well as conductivity, the two measurements are strongly related. The term “total dissolved solids” is often used for salinity. In this study, we will refer to water hardness and electrical conductivity.

The majority of the world’s population lives in areas where mosquitoes are present, and the worldwide incidence of mosquito-borne diseases is enormous. *Aedes aegypti* (Linnaeus, 1762) and *Ae. albopictus* (Skuse, 1894) are invasive species and continue to expand their distribution range. They are very efficient to transmit several viruses between vertebrate hosts causing deadly diseases such as dengue, chikungunya, yellow fever, West Nile fever and Zika [19]. Expansion of the transmission season in endemic areas, re-emergence in certain areas after a prolonged absence of transmission, spread to areas where transmission had not previously occurred, and outbreaks of these diseases are becoming more frequent in both developed and developing countries. With limited commercially available vaccines and antiviral therapies, *Aedes* spp. populations control is a cornerstone to prevent disease transmission. Since the world has observed the outbreak of Zika in the Americas in late 2015, there are renewed interests globally, to use the sterile insect technique (SIT) as part of area wide integrated pest management (AW-IPM) programmes to control mosquito-borne diseases [9,10,20,21,22,23]. The SIT relies on the mass production of mosquitoes, which demands a huge amount of water [24,25]. However, obtaining sufficient water of reliable quality is still challenging due to aridity, lack of environmental protection and adequate treatment techniques, especially in developing and least developed countries, where access even to clean drinking water is limited [26]. *Aedes* mosquitoes are being mass-reared for release in disease control programs around the world. While researchers strive to provide optimum rearing conditions and are establishing standard operating procedures [27,28], water quality can differ between countries and seasons [29,30]. In response to variations in the mass-rearing outputs in many Food and Agriculture Organization of the United Nations (FAO) and International Atomic Energy Agency (IAEA) Member States, there is an urgent need to investigate the effect of water quality on mosquito development, productivity and resulting adult quality. Recent evidence demonstrated the influence of water hardness on the development of *Anopheles* [31] and *Culex* [32] mosquitoes. However, to the best of our knowledge, the impact of the TW quality (in relation to hardness or electrical conductivity) on *Aedes* mosquito species is insufficiently documented. In this study, we aim to explore the respective responses of *Ae. aegypti* and *Ae. albopictus* to different TW hardness and electrical conductivity levels (ROW, TW and mixtures of varying proportions of ROW and TW). Parameters assessed include time to pupation, pupation rate, emergence rate, adult production rate, adult body size and adult longevity.

## 2. Materials and Methods

### 2.1. Source of Experimental Mosquitoes

In this study, we used colonies of *Ae. aegypti* and *Ae. albopictus* originating from Juazeiro, Brazil (provided by Biofabrica Moscamed, IAEA Collaborative Center since 2012) and Italy (provided by Centro Agricoltura Ambiente, IAEA Collaborative Center since 2018), respectively. They were established and maintained at the Insect Pest Control Laboratory (IPCL) under controlled environmental conditions: the larval rearing room was maintained at 28 ± 2 °C, 80 ± 10% RH and the adult rearing room at 26 ± 2 °C, 60 ± 10% RH, with a 14:10 h light:dark (L:D) cycle with 1 h periods of simulated dusk and dawn in both rooms. *Aedes aegypti* and *Ae. albopictus* eggs used in these experiments were obtained following mass-rearing procedures developed at the IPCL [28,33,34,35].

### 2.2. Preparation of Water Media and Determination of Their Hardness/Electrical Conductivity and pH

Water media (*n* = 5) with increasing hardness or electrical conductivity were prepared for tests by adding TW to ROW as follows: (1)100% ROW, (2) 80% ROW + 20% TW, (3) 50% ROW + 50% TW, (4) 20% ROW + 80% TW, (5) 100% TW. The ROW water was considered as the baseline water hardness level and the control treatment as it represents water routinely and successfully used for rearing *Aedes* mosquitoes. TW was considered as the highest level of hardness in this experiment. After dilution in large containers, four samples of each water treatment were taken to determine the hardness and the conductivity values. The remaining water was used for rearing.

The water hardness was measured using the Dosatest^®^ test strips which is a semi-quantitative method. Clear colour changes from green to red ensure reliable results within seconds. The strip was simply and properly dipped and the colour compared with the colour chart provided on the bottle with range values indicated in mmol/m^3^; values expressed in mmol/m^3^ were later converted into mg/L CaCO_3_ following the formula: 1 mmol/m^3^ = 10 °f = 5.60 °d = 7022 °e = 100.09 mg/l CaCO_3_ (°f = degrees French hardness, °d = degrees German hardness), °e = degrees Clark hardness).

The electrical conductivity was measured using Go Direct^®^ Conductivity Probe (Vernier Go Direct^®^, 13,979 SW Millikan Way Beaverton, OR, USA) with a range of 0 to 20,000 μS/cm. It connects via Bluetooth^®^ wireless technology or via USB to the electronic device (computer or telephone). Dosatest^®^ test strips and electrical conductivity measurements were carried out for each experiment and thus twice in this study and gave similar results. The pH values were measured using a pH meter (WTW pH 3110, Xylem Analytics, Weilheim, Germany).

### 2.3. Assessment of the Effects of Water Treatments on Larval Development and Adult Quality

The five water treatments described above (1) 100% ROW, (2) 80% ROW + 20% TW, (3) 50% ROW + 50% TW, (4) 20% ROW + 80% TW, (5) 100% TW were applied to both species.

For each species, eggs were hatched in glass jars overnight following standard procedures developed at the IPCL [28,33,34,35]. After hatching, batches of 200 first-instar larvae were manually counted and haphazardly allocated to the different water media prepared. A total of 8000 first-instar were used for each experiment. Larvae were reared in transparent plastic containers (L × W × H = 150 × 90 × 50 mm) and filled with 500 mL of rearing medium. The IAEA black soldier fly-based-diet (4% (vol/wt) which consists of 50% tuna meal + 15% brewer’s yeast + 35% black soldier fly larvae powder [34,36] was used with the following daily amounts: 5 mL on day 1, 10 mL on day 2, 20 mL on day 3, 10 mL on day 6. Four replicates were performed for each water treatment and the experiment was carried out twice for each species. Larvae were checked daily for pupation, and pupae were collected and counted on a daily basis. For all experimental water treatments, we recorded: (i) time to pupation (the number of days from hatching to pupation), (ii) pupation rate, (iii) emergence rate, (iv) male and female body size: after emergence, 20 females and 20 males per treatment (5 per replicate) were randomly selected and the right wings detached and mounted on glass microscope slides under a cover slip. A photograph of each wing was taken under a dissecting microscope (Leica MZ16 FA, Leica Microsystems (Switzerland) Ltd, Heerbrugg, Switzerland.). Wing length was measured from the tip of the wing (excluding fringe) to the distal end of the alula using analySIS^®^FIVE software. Wing length is considered to be a proxy for mosquito body size, (v) male and female longevity: 40 males and 40 females that emerged the same day (10 per replicate) from each water treatment were transferred and maintained in a cage separately (15 × 15 × 15 cm, Bugdorm.com, Taichung, Taiwan) for measurement of longevity. A 10% sugar solution was supplied in a 150-mL plastic bottle containing a sponge and mortality was recorded daily. For the longevity monitoring, adults were maintained at 28 ± 2 °C, 80 ± 10% RH and 14:10 h photoperiod.

### 2.4. Assessment of the Effects of Water Treatments on Larval Development and Production with Low Food Quantity

Based on the variable and low pupation rates obtained in the previous experiment, and in order to verify that the resulting effects were caused only by water treatments, a second experiment was conducted. The amount of daily food provided to larvae was halved to give 5 mL on day 1, 5 mL on day 2, 10 mL on day 3, 5 mL on day 6. Subsequently, the effect of different water treatments (as described in the experiment above) on larval development was assessed. Four replicates for each water treatment were performed. Time to pupation, pupation and the emergence rates were assessed and compared to experiment 1.

### 2.5. Statistical Analysis

Statistical analyses were performed using R Software version 3.5.2 (R Development Core Team 2008, URL http://www.R-project.org/). A Gaussian linear mixed-effects model was used with time to pupation, male and female body size assigned as response variables, water media as a fixed effect and replicate as a random effect [37]. We also used binomial generalized linear mixed models fit by maximum likelihood (Laplace Approximation) with pupation rate, emergence rate and adult as response variables, water media as fixed effect and the replicate as a random effect. The full models were checked for overdispersion using Bolker’s function for validation. The longevity of mosquitoes was analysed using Kaplan-Meier survival analyses using GraphPad Prism v.5.0 ((Windows, Graphpad Software, La Jolla, CA, USA; www.graphpad.com). The log-rank (Mantel-Cox) test was used to compare the level of survival between different treatments. The Bonferroni correction method was applied for each pair of groups to account for the multiplicity comparisons.

## 3. Results

### 3.1. Hardness, Electrical Conductivity and pH of the Rearing Media

Hardness, electrical conductivity and pH of the rearing media are presented in Table 1. Water hardness values were notably different between water media, ranging from 0 to 400.36 mg/L CaCO_3_. EC ranged from 11.04 ± 0.01 to 686.50 ± 0.23 µS/cm and pH from 5.85 ± 0.005 to 7.32 ± 0.006. Based on standard classification of water hardness as described by the World Health Organization (WHO) [38], our rearing media can be classified as soft (100% ROW), moderately hard water (80% ROW + 20% TW), hard water (50% ROW + 50% TW), and very hard water (20% ROW + 80% TW and 100% TW).

### 3.2. Effects of Water Treatments on Time to Pupation

**Figure 1:**
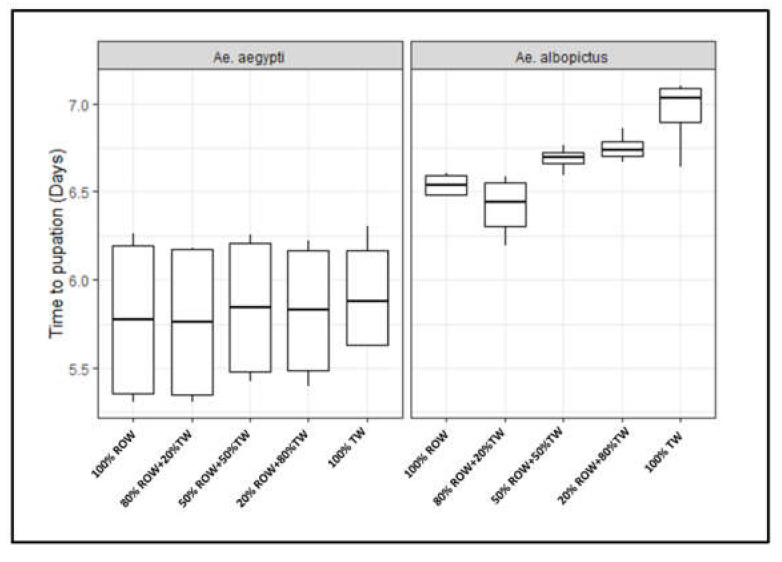
Time to pupation of *Aedes aegypti* and *Aedes albopictus* reared at different hardness/electrical conductivity levels of larval rearing water. As water hardness/electrical conductivity level increased, time to pupation gradually increased. Each box denotes the median as a line across the middle, the quartiles (25th and 75th percentiles), the minimum and maximum values at the ends of the vertical lines. Results are expressed as mean ± SE. ROW = reverse osmosis water, TW = tap water.

Time to pupation was affected by the level of water hardness in both species (Figure 1). As water hardness level increased, time to pupation gradually increased (i.e., delayed development at higher hardness levels), with cohorts reared at the highest hardness levels spending the longest time as immature. In *Ae. aegypti*, time to pupation in water treatments 80% ROW + 20% TW, 50% ROW + 50% TW and 20% ROW + 80% TW did not differ with the control treatment, although they were slightly increased (Table S1). However, time to pupation in the 100% TW was significantly increased compared to control treatment (df = 12, t = 2.66, *p* = 0.021). In *Ae. albopictus*, time to pupation in the treatments 20% ROW + 80% TW and 100% TW were significantly higher than the control treatment (Table S2, df = 12, *t* = 2.65, *p* = 0.021 and df = 12, *t* = 5.14, *p* ˂ 0.001, respectively). Interestingly, with this feeding regime, whatever the water hardness treatment, the time to pupation was significantly higher in *Ae. albopictus* than *Ae. aegypti* (df = 25, *t* = 4.11, *p* ˂ 0.001).

### 3.3. Effects of Water Treatments on Pupal Production, Emergence Rate and Adult Production

In *Ae. albopictus*, pupae production significantly decreased with increasing water hardness as compared to the control medium (Table S2, *p* ˂ 0.05). In *Ae. aegypti*, pupation rate was negatively affected by water hardness ranging from 140.126 to 400.36 mg/CaCO_3_ i.e., the water media 50% ROW + 50% TW to 100% TW (Table S1). In both species, the emergence rates were not significantly different between water treatments (Tables S1 and S2). Consequently, adult production was negatively affected in both species, similarly to the pupation rate (Tables S1 and S2).

### 3.4. Effects of Water Treatments on Adult Body Size

**Figure 2:**
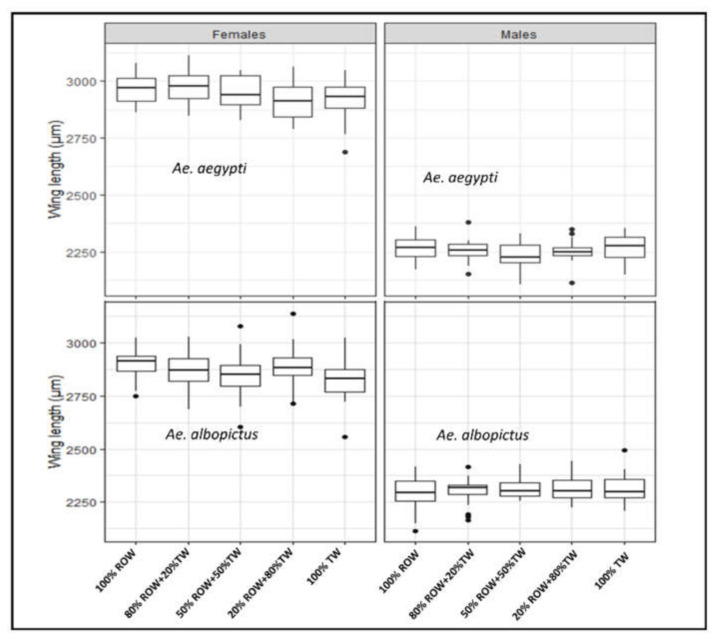
Body size of male and female *Aedes aegypti* and *Aedes albopictus* reared at different hardness/electrical conductivity levels of larval rearing water. Each box denotes the median as a line across the middle, the quartiles (25th and 75th percentiles), the minimum and maximum values at the ends of the vertical lines. Results are expressed as mean ± SE. ROW = reverse osmosis water, TW = tap water.

In comparison to the control medium, the media 20% ROW + 80% TW (*t* = −2.151, df = 92, *p* = 0.034) and 100% TW (*t* = −2.192, df = 92, *p* = 0.031) significantly decreased female body size (Figure 2) in *Ae. aegypti*. In *Ae. albopictus*, the media 50% ROW + 50% TW (*t* = −2.073, df = 92, *p* = 0.041) and 100% TW (*t* = −2.715, df = 92, *p* = 0.008) significantly decreased female body size. No significant effect was found in male body size in either species (Figure 2, *p* > 0.05).

### 3.5. Effects of Water Treatments on Adult Longevity

**Figure 3:**
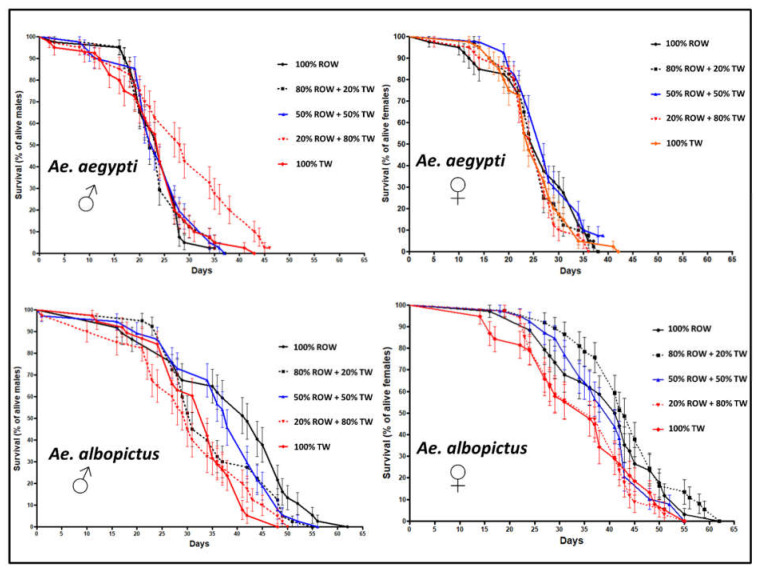
Longevity of male and female *Aedes aegypti* and *Aedes albopictus* reared in different hardness/electrical conductivity levels of larval rearing water. ROW = reverse osmosis water, TW = tap water.

The survival curves, the mean and median survival durations of males and females reared with different water media are presented in Figure 3 and Table 2. Overall, in *Ae. aegypti*, the longevity of males and females was not affected negatively by the water hardness level compared to the control medium (graphical observation, Figure 3, Log-rank (Mantel-Cox) test, *p* > 0.005). However, the longevity of males was higher when reared in the water medium 20% ROW + 80% TW (Log-rank (Mantel-Cox) test, χ*^2^* = 11.25, df = 1, *p* ˂ 0.001) as compared to the control.

In *Ae. albopictus*, there was a significant variation in longevity of males and females between water treatments (Log-rank (Mantel-Cox) test, χ*^2^* = 17.31, df = 4, *p* = 0.002 and Log-rank (Mantel-Cox) test, χ*^2^* = 12.76, df = 4, *p* = 0.01 for males and females, respectively). Compared to the control, increased water hardness decreased the longevity of adult males (Figure 3, Log-rank (Mantel-Cox) test, χ*^2^* = 11.66, df = 1, *p* ˂ 0.001 and Log-rank (Mantel-Cox) test, χ*^2^* = 14.41, df = 1, *p* ˂ 0.001 for the media 20% ROW + 80% TW and 100% TW respectively). Moreover, in females, the longevity decreased in media 20% ROW + 80% TW and 100% TW in comparison to the medium 80% ROW + 20% TW (Log-rank (Mantel-Cox) test, χ*^2^* = 11.7, df = 1, *p* ˂ 0.001 and Log-rank (Mantel-Cox) test, χ*^2^* = 9.86, df = 1, *p* = 0.002). Whatever the rearing medium, *Ae. albopictus* survived longer than *Ae. aegypti* (Figure 3).

### 3.6. Effects of Water Treatments on Time to Pupation, Pupation and Emergence Rates at Low Feeding Amounts

In the second experiment with low larval food quantities, time to pupation gradually increased with increased water hardness in *Ae. albopictus*. The treatments 20% ROW + 80% TW and 100% showed a significant increase in time to pupation in comparison to the control treatment 100% ROW (Table 3, df = 11, *t* = 3.82, *p* = 0.003 and df = 11, *t* = 3.39, *p* = 0.006, respectively), consistent with first experiment. No significant difference was observed in *Ae. aegypti*, although there was a trend for increased time to pupation. As compared to the control treatment 100% ROW, pupae production significantly decreased in all water treatments whatever the species, consistent with results obtained in experiment 1. However, with this feeding regime, the pupation rate was slightly higher (89.88 ± 1.19%) in *Ae. albopictus*, but not in *Ae. aegypti* (77.93 ± 3.74%), as compared to the previous experiment. No difference was observed in emergence rates between water treatments in both species as shown in experiment 1.

## 4. Discussion

The ionic composition of water can be critical for the development and survival of aquatic organisms and every organism has a typical range that it can tolerate. Despite the plethora of information on the physico-chemical properties of the larval habitats, including pH, temperature, humidity, resource availability and larval crowding as key factors in determining the presence, development, survival and population dynamics and distribution of mosquitoes [3,39,40,41,42,43], little or no work has been done on the isolated or specific effects of water hardness/electrical conductivity, pH on *Aedes* mosquito’s life-history traits. This investigation was undertaken to evaluate the tolerance of *Ae. aegypti* and *Ae. albopictus* mosquitoes to variations of water quality in relation to hardness, electrical conductivity and pH, and to thereby determine whether hard water (generally TW) can be a suitable medium for rearing *Aedes* mosquitoes in laboratory settings. Data obtained in the present study showed that TW quality had measurable effects on the development and quality of *Aedes* mosquito species. Indeed, results showed that the increase in water hardness/electrical conductivity level increased the average larval development time in both species. Slower larval development was observed in mosquitoes reared at higher water hardness/electrical conductivity levels. This suggests that depending on the quantity, ions in the aquatic environment may affect the growth and the metabolism during moulting of the larvae, and thus the speed and extent of their development. Similar results have been found in *Cx quinquefasciatus*. The duration of the development of this species gradually increases as water hardness/electrical conductivity levels increase [32]. Furthermore, the present study revealed a reduction in pupation rate with increasing hardness/electrical conductivity levels, particularly in *Ae. albopictus*, indicating significant larval mortality. It is worth mentioning that water media with high levels of hardness/electrical conductivity were prone to scum (biofilm) formation on water surface during rearing, which can lead to fouling and ultimately to increased mortality or inferior adults especially in case of excess amounts of food (overfeeding). This suggests that ion content in the water might affect the microbial/bacterial community in the diet and in the water mix over time and therefore the growth of larvae due to a reduced availability of nutriments caused by bacterial competition.

In this experiment, male longevity was negatively impacted by high levels of water hardness/electrical conductivity in *Ae. albopictus*. Akpodiete et al. [31] found that different strains of *An. Gambiae* showed a longer development time, higher larval survival and smaller body size when reared with deionized water as compared to mineral water. However, the conductivity and hardness conditions of this mineral water are low and are representative of the second level of hardness of this study, i.e., 80% ROW + 20% TW or moderately hard water. Body size, along with longevity, is among the valuable indicators of insect quality [44], and is therefore crucial for the success of any male release programme. Small size will likely lead to poor performance in the field. In some insects, such as tephritid fruit flies, it has been demonstrated that insects that completed larval development tend to more rapidly become larger and are of higher quality than those that developed more slowly [45]. It has also been shown that female body size correlates with fecundity [46], as large females are more likely to ingest a larger volume of blood than small ones, and therefore successfully oviposit and lay more eggs. For any male release programme, if the longevity is reduced, the number of males to be released should be increased.

Although negative effects of water hardness/electrical conductivity were observed in both species, these results have demonstrated the potential of these mosquito species to exhibit some degree of tolerance to water hardness/electrical conductivity. For example, Ramasamy et al. [47] reported that *Ae. aegypti* and *Ae. albopictus* have successfully exploited brackish water collections in unused wells and discarded artificial containers of up to 15 ppt salinity in the peri-urban environment to oviposit and undergo preimaginal development. Although hardness, conductivity and salinity are not exactly the same, salinity as a measure of the amount of salts in the water may have other impacts on mosquito life cycle to a greater extent due to the presence of sodium chloride. Because dissolved ions increase salinity as well as conductivity, the two measures are strongly correlated. However, every organism has a typical hardness/electrical conductivity range that it can tolerate. *Aedes albopictus* was found to be more susceptible to increasing water hardness/electrical conductivity than *Ae. aegypti,* underlining differences between these species, although they coexist throughout most of their geographical distribution. In natural environments, *Ae. aegypti* and *Ae. albopictus* are thought to differ only subtly in their preferred larval breeding sites. The lower adaptive capacity of *Ae. albopictus* found in this study is somewhat surprising, given that it was demonstrated that this species has higher survivorship than *Ae. aegypti* in the laboratory (this study and [48]). Additionally, its superior larval competitive ability has been proposed as a reason to explain the recent displacement of *Ae. aegypti* by *Ae. albopictus* in parts of the southeastern U.S. [19,49]. However, Wigglesworth [50] showed that larvae of *Ae. aegypti* and *Cx pipiens* can osmoregulate and ionoregulate very effectively in essentially all media more diluted than their haemolymph by producing a diluted urine to get rid of water and replace lost salts by active ion uptake through the cuticle.

Potential ions present in the TW include calcium (Ca^2+^), magnesium (Mg^2+^), sodium (Na^+^), potassium (K^+^), chloride (Cl^−^), nitrate (NO_3_^−^), sulfate (SO_4_^2−^), bicarbonate (HCO_3_^−^), fluoride, lead, and zinc [51]. Water ions have a beneficial concentration range above which they may have an adverse effect [52]. The physiological mechanisms which may account for this effect in mosquitoes are not well understood, and are beyond the scope of this study. However, insects exposed to salty environments are generally challenged by osmotic stresses. In aqueous environments, larval survival depends on the ability to regulate the hydromineral balance of the haemolymph to maintain homeostasis [53]. In this study, presumably, insects exposed to increased water hardness/electrical conductivity might have faced a considerable osmoregulatory challenge as many organisms like marine osmoconformers lacking the capacity to regulate osmolarity and the ion content of their internal fluids. It is likely that excess ions derived from ingestion create problems for the maintenance of homeostasis. High ingestion of ions through the high rate of drinking water has been demonstrated in *Ae. taeniorhychus* [54,55]. On the other hand, knowing that the cuticle of fresh-water species is more permeable to water than that of saline-water mosquito larvae [56], high water hardness could increase the permeability to ions, increasing their respective effluxes and, potentially, larval mortality. Osmoconformation and osmoregulation are well known as regulatory mechanisms for dealing with ionic environments in aquatic organisms [57]. Kengne et al. [58] showed that both *Ae. aegypti* and *Ae. albopictus* are hyper osmoregulators. It has also been shown that *An. gambiae* mosquitoes can adjust their biological program through proteome changes to counter heavy metal pollution [59]. Higher salinity tolerance in the *Enochrus* species was also associated with an increase in the relative abundance of branched alkanes (cuticule hydrocarbons) [60] or overexpression of ions channels aquaporines (osmoregulation) [61]. However, the mechanisms of water hardness effects or tolerance need to be further elucidated. Knowledge of rearing water quality and its impact on mosquito development (from the results of this study) have clear applied relevance, as the success of the sterile insect technique depends critically on the number and quality of mass-produced and released males. Mineral levels of TW vary among countries, and even among different water sources. Yasin et al. [51] reported the electrical conductivity of TW from Ethiopia was 366.93 µS/cm, which is almost 50% lower than the value of the TW used in this study, and which correspond to the mix of 50% ROW + 50% TW. With regard to the results of this study, TW from Ethiopia is more suitable for rearing *Aedes* mosquitoes than TW in Austria. It is, therefore, of interest to evaluate the quality (in relation to hardness or electrical conductivity) of the rearing water before its use for rearing *Aedes* mosquitoes. In a recent SIT experiment organized in Brazil, TW had such a negative impact on the survival of *Ae. aegypti* larvae that mineral water had to be purchased [62]. Further studies, including flight ability, fecundity, and fertility in mass-rearing conditions, are needed to elucidate the impact of water hardness in SIT and other related techniques, including Wolbachia-based and transgenic approaches.

Although this research was designed to answer practical questions about the use of TW for rearing *Aedes* mosquitoes and achieved this goal, there were some limitations and shortcomings. The fact that TW can differ from the ROW in many other factors, together with the limited range of variables measured (three), represents a potential bias that may interact with the specific effect of the hardness or electrical conductivity. In experiment 1 with higher larval food quantities, we found variable pupation rates including rates falling below the expected rates generally observed in most routine rearing conditions. In the second experiment with a reduced food quantity (half of the initial amount), there was a slight increase in pupation rates. Whatever the feeding regimes used in this study, there was evidence of the negative effect of tap water on rearing outputs. However, care should be taken regarding food quantities delivered to larvae to avoid negative effects on outputs.

## 5. Conclusions

Water quality is a factor of great importance in the larval environment of mosquito species. Increasing hardness/electrical conductivity level beyond 140 mg/L CaCO_3_ (or 368 µS/cm) was found to be a limiting factor, as it influenced time to pupation, pupation rate, body size and longevity of *Ae. aegypti* and *Ae. albopictus*. While ROW is highly recommended, with respect to cost-effective methods for improved mass-rearing toward SIT application, TW or a mix of TW with ROW up to certain limit of water hardness/electrical conductivity could provide adequate conditions for rearing these two mosquito species. Differences in the ability to maintain homeostatic control of water and ion balance may explain large parts of the observed interspecific variation. These results may have important implications for the implementation of the SIT in areas where ROW is a scarce or costly resource. For any other source of water, characteristics such as hardness, electrical conductivity and pH should be considered when using water for rearing mosquitoes for release purposes in order to optimize the production performance of mass-rearing facilities.

## Figures and Tables

**Table 1:** Measured hardness, conductivity and pH of the water media used for rearing *Aedes* mosquitoes in the present experiment. Conductivity and pH values are expressed as mean ± SE. ROW = reverse osmosis water, TW = tap water.

Parameters		100% ROW	80%ROW + 20%TW	50%ROW + 50%TW	20%ROW + 80%TW	100% TW
Hardness Dosatest^®^ hardness test strips	(mmol/m^3^)	0–0.3	0.7–1.2	1.4–2.5	2.8–3.7	3.7–4
	(mg/L CaCO_3_)	0–30.03	70.06–120.11	140.13–250.23	280.25–370.33	370.33–400.36
Conductivity	(µS/cm)	11.04 ± 0.01	154.53 ± 0.10	368.45 ± 0.14	557.83 ± 0.17	686.50 ± 0.23
pH		5.85 ± 0.005	6.82 ± 0.005	7.09 ± 0.005	7.24 ± 0.006	7.32 ± 0.006

**Table 2:** Mean ± se (days) and median survival (days) of *Aedes aegypti* and *Aedes albopictus* males and females reared under different water hardness treatments. ROW = reverse osmosis water, TW = tap water.

Species	Sex	Parameters	100% ROW	80%ROW + 20%TW	50%ROW + 50%TW	20%ROW + 80%TW	100% TW
***Ae. aegypti***	Males	Mean	23.05 ± 3.12	23.68 ± 3.28	23.88 ± 4.06	28.08 ± 6.47	23.05 ± 4.91
		Median	24	22	23	28.5	24
	Females	Mean	25.50 ± 4.75	25.73 ± 3.43	26.86 ± 3.25	24.70 ± 3.64	25.25 ± 3.85
		Median	27	27	27	27	24
***Ae. albopictus***	Males	Mean	39.00 ± 7.36	34.90 ± 4.88	36.70 ± 6.50	30.00 ± 6.35	32.21 ± 4.92
		Median	42	31	38	30	34
	Females	Mean	39.50 ± 6.43	43.51 ± 5.70	38.90 ± 4.84	35.24 ± 5.60	34.21 ± 6.65
		Median	41.5	43	41	37	36

**Table 3:** Mean time to pupation, pupation and emergence percentages (mean± se) in *Aedes aegypti* and *Aedes albopictus* reared under different water hardness treatments. ROW = reverse osmosis water, TW = tap water. Within a row, different letters with the control treatment (100% ROW) indicate a statistically significant difference (*p* < 0.05).

Species		100% ROW	80%ROW + 20%TW	50%ROW + 50%TW	20%ROW + 80%TW	100% TW
*Aedes aegypti*	Time to pupation	7.09 ± 0.08 ^a^	7.22 ± 0.04 ^a^	7.26 ± 0.08 ^a^	7.22 ± 0.08 ^a^	7.04 ± 0.15 ^a^
	Pupation %	77.93 ± 3.74 ^a^	62.56 ± 712 ^b^	64.63 ± 3.67 ^b^	51.06 ± 6.26 ^b^	52.38 ± 6.22 ^b^
	Emergence %	98.48 ± 0.42 ^a^	98.92 ± 0.40 ^a^	98.50 ± 0.30 ^a^	98.36 ± 0.37 ^a^	98.68 ± 0.41 ^a^
*Aedes albopictus*	Time to pupation	7.97 ± 0.05 ^a^	8.06 ± 0.03 ^a^	7.94 ± 0.01 ^a^	8.24 ± 0.04 ^b^	8.21 ± 0.11 ^b^
	Pupation %	89.88 ± 1.19 ^a^	87.13 ± 1.30 ^b^	83.67 ± 1.48 ^b^	83.75 ± 2.09 ^b^	74.00 ± 9.26 ^b^
	Emergence %	99.01 ± 0.85 ^a^	99.51 ± 0.33 ^a^	98.53 ± 1.70 ^a^	99.46 ± 0.36 ^a^	99.51 ± 0.34 ^a^

## Data Availability

All data generated or analysed during this study are included in this published article.

## Supplementary Materials

The following are available online at https://www.mdpi.com/2075-4450/12/1/57/s1, Table S1: Results of linear mixed models and binomial generalized linear mixed models for the effect of water hardness/electrical conductivity on *Aedes aegypti* life history trait parameters. Table S2: Results of linear mixed models and binomial generalized linear mixed models for the effect of water hardness/electrical conductivity on *Aedes albopictus* life history trait parameters.
