# Supplementary material for: Does Tap Water Quality Compromise the Production of Aedes Mosquitoes in Genetic Control Projects?
Source: Insects. 2021 Jan 12;12(1):57. doi: 10.3390/insects12010057 (PMC7826741; doi:10.3390/insects12010057)
Supplement: Supplementary file 1 [file insects-12-00057-s001.pdf]

Table S1. Results of linear mixed models and binomial generalized linear mixed models for the effect of water hardness/electrical conductivity on *Aedes aegypti* life history trait parameters. Values were compared to the control 100% ROW. ROW = reverse osmosis water, TW = tap water.

Species	Parameters		Value	SE	DF	t-value	p-value
		**Intercept**	5.78	0.22	12	26.04	**<0.001**
		**80%ROW+20%TW**	-0.03	0.05	12	-0.50	0.63
	**Time to pupation**	**50%ROW+50%TW**	0.06	0.05	12	1.18	0.26
		**20%ROW+80%TW**	0.04	0.05	12	0.77	0.45
		**100%TW**	0.14	0.05	12	2.66	**0.02**
		**Intercept**	2265.56	12.17	92	186.19	**0.00**
		**80%ROW+20%TW**	-4.77	17.21	92	-0.28	0.78
	**Male body size**	**50%ROW+50%TW**	-26.88	17.21	92	-1.56	0.12
		**20%ROW+80%TW**	-10.47	17.21	92	-0.61	0.54
		**100%TW**	7.03	17.21	92	0.41	0.68
		**Intercept**	2963.92	16.52	92	179.39	**0.00**
		**80%ROW+20%TW**	14.06	23.37	92	0.60	0.54
	**Female body size**	**50%ROW+50%TW**	-14.57	23.37	92	-0.62	0.53
		**20%ROW+80%TW**	-50.27	23.37	92	-2.15	**0.03**
		**100%TW**	-51.22	23.37	92	-2.19	**0.03**
Aedes aegypti			**Estimate**	**SE**		**z-value**	**p-value**
		**Intercept**	0.90	0.36		2.52	**0.01**
	**Pupation rate**	**80%ROW+20%TW**	0.19	0.47		0.40	0.69
		**50%ROW+50%TW**	-1.42	0.47		-3.01	**0.003**
		**20%ROW+80%TW**	-2.12	0.47		-4.48	**7.32e-6**
		**100%TW**	-1.24	0.47		-2.62	**0.01**
		**Intercept**	3.95	0.26		15.14	**<2e-16**
		**80%ROW+20%TW**	0.89	0.42		2.09	**0.04**
	**Emergence rate**	**50%ROW+50%TW**	0.26	0.42		0.61	0.54
		**20%ROW+80%TW**	0.91	0.63		1.43	0.15
		**100%TW**	0.39	0.42		0.93	0.35
		**Intercept**	0.62	0.11		5.59	**2.33e-08**
		**80%ROW+20%TW**	0.27	0.08		3.33	**0.0008**
	**Adult production rate**	**50%ROW+50%TW**	-1.12	0.08		-13.94	**< 2e-16**
		**20%ROW+80%TW**	-1.77	0.08		-20.91	**< 2e-16**
		**100%TW**	-0.89	0.08		-11.23	**< 2e-16**

Table S2. Results of linear mixed models and binomial generalized linear mixed models for the effect of water hardness/electrical conductivity on *Aedes albopictus* life history trait parameters. Values were compared to the control 100% ROW. ROW = reverse osmosis water, TW = tap water.

Species	Parameters		Value	SE	DF	t-value	p-value
		**Intercept**	6.54	0.07	12	95.15	**<0.0001**
		**80%ROW+20%TW**	-0.13	0.08	12	-1.57	0.14
	**Time to pupation**	**50%ROW+50%TW**	0.15	0.08	12	1.83	**0.09**
		**20%ROW+80%TW**	0.21	0.08	12	2.65	**0.02**
		**100%TW**	0.41	0.08	12	5.14	**0.0002**
		**Intercept**	2292.63	14.57	92	157.31	**0.00**
		**80%ROW+20%TW**	9.31	20.61	92	0.45	0.65
	**Male body size**	**50%ROW+50%TW**	16.06	20.61	92	0.78	0.44
		**20%ROW+80%TW**	21.33	20.61	92	1.03	0.30
		**100%TW**	22.96	20.61	92	1.11	0.27
		**Intercept**	2908.22	20.78	92	139.94	**0.00**
		**80%ROW+20%TW**	-27.63	29.39	92	-0.94	0.35
	**Female body size**	**50%ROW+50%TW**	-60.93	29.39	92	-2.07	**0.04**
		**20%ROW+80%TW**	-21.75	29.39	92	-0.74	0.46
		**100%TW**	-79.79	29.39	92	-2.72	**0.008**
Aedes albopictus							
			**Estimate**	**SE**		**z-value**	**p-value**
		**Intercept**	1.05	0.29		3.50	**<0.001**
		**80%ROW+20%TW**	-0.64	0.42		-1.49	0.14
	**Pupation rate**	**50%ROW+50%TW**	-1.15	0.43		-2.70	**0.01**
		**20%ROW+80%TW**	-1.69	0.43		-3.95	**7.71e-05**
		**100%TW**	-1.85	0.43		-4.33	**1.50e-05**
		**Intercept**	4.14	0.21		19.28	**<2e-16**
		**80%ROW+20%TW**	0.17	0.36		0.48	0.63
	**Emergence rate**	**50%ROW+50%TW**	-0.17	0.34		-0.49	0.63
		**20%ROW+80%TW**	0.002	0.40		0.006	0.99
		**100%TW**	-0.099	0.40		-0.25	0.80
		**Intercept**	0.83	0.15		5.41	**6.26e-08**
		**80%ROW+20%TW**	-0.58	0.07		-8.13	**4.24e-16**
	**Adult production rate**	**50%ROW+50%TW**	-0.97	0.07		-13.74	**< 2e-16**
		**20%ROW+80%TW**	-1.44	0.07		-19.93	**< 2e-16**
		**100%TW**	-1.60	0.07		-21.80	**< 2e-16**
